# Spatio-temporal occurrence and habitat characteristics of *Aedes aegypti* (Diptera: Culicidae) larvae in Southern Afar region, Ethiopia

**DOI:** 10.1186/s41182-024-00612-5

**Published:** 2024-08-02

**Authors:** Mohammed Seid, Esayas Aklilu, Abebe Animut

**Affiliations:** 1 Aklilu Lemma Institute of Pathobiology, Addis Ababa University, Addis Ababa, Ethiopia; 2 Department of Biology, College of Natural and Computational Sciences, Mattu University, Mattu, Ethiopia

**Keywords:** *Aedes aegypti*, Afar Region, Ethiopia, Habitat characteristics, Occurrence

## Abstract

**Background:**

Describing spatio-temporal occurrence and habitat characteristics of *Aedes* mosquito larvae is crucial for the control of *Aedes* borne viral diseases. This study assessed spatio-temporal abundance and habitat characteristics of *Aedes* larvae in the Southern Afar Region, Ethiopia.

**Methods:**

Immature mosquitoes were surveyed in Awash Sebat, Awash Arba, and Werer towns of the Southern Afar Region once per month from May 2022 to April 2023. Larvae and pupae surveys were carried out along the available water-holding containers. The collected larvae/pupae were reared to adults and identified by  species/genus morphologically. The physical and chemical properties of the habitats were also characterized.

**Results:**

A total of 9099 *Aedes* larvae/pupae were collected, of which 53.6% (4875) were from Awash Sebat, 29.5% (2687) from Awash Arba and 16.9% (1537) from Werer. Water-holding tyres harboured the highest number of *Aedes* larvae/pupae followed by water-storage drums. All the *Aedes* larvae/pupae reared to adults were morphologically identified as *Aedes aegypti*. The overall Container Index was 47.28%, House Index 18.19%, Breteau Index 59.94% and Pupal Index 171.94. Significant positive relations were observed in the occurrences of *Ae. aegypti* larvae/pupae with water-holding tyre (AOR = 15.89, CI = 3.55–71.09, *p* < 0.001), water storage drums (AOR = 19.84, CI = 4.64–84.89, *p* < 0.001), domestic habitat (AOR = 3.76, CI = 1.27–11.12, *p* = 0.017), and significant negative relations were observed with *Ae. aegypti* larvae/pupae occurrence and tap water source (AOR = 0.08, CI = 0.02–0.31, *p* = 0.001). *Ae. aegypti* larvae/pupae densities showed positive relations with dissolved oxygen (β = 0.523, *p* < 0.001) and total hardness (β = 0.475, *p* = 0.034) of water.

**Conclusions:**

Diverse types of artificial water-holding containers were positive for *Ae. aegypti* larvae/pupae. *Ae. aegypti* larvae/pupae were abundant in used water-holding tyres, water storage drums, and cement tanks in Awash Sebat, Awash Arba, and Werer towns. This could  put the residents of the towns at high risk of infections with *Ae. aegypti* transmitted viral diseases such as chikungunya and dengue outbreaks. Thus, we recommend artificial water-holding container management as a strategy to control *Ae. aegypti* and hence the arboviral diseases transmission.

**Supplementary Information:**

The online version contains supplementary material available at 10.1186/s41182-024-00612-5.

## Introduction

Human infections associated with *Aedes* mosquito-transmitted viruses such as dengue virus (DNV), yellow fever virus (YFV) chikungunaya (CHIKV), and zika virus (ZIKV) are spreading at an alarming rate. They exert a huge burden on populations, health systems, and economies in tropical countries [1]. Dengue fever alone is endemic in more than 125 countries, and the number and geographic distribution of the cases have increased significantly in recent years [2]. Africa is considered as an epicentre for the emergence and re-emergence of life threatening arboviruses such as DNV and CHIKV particularly in East African countries [3]. They have dramatically increased over the last two decades [4]. Several arboviral infection outbreaks have been reported from various parts of Ethiopia since the 1960s. These include dengue fever outbreaks in Gewane district of Afar Region [5], yellow fever in South Omo [6], dengue and chikungunya in Dire Dawa city administration and Somali Region [6–8].

Determinants of the increasing trend of the pathogenic arboviruses and their mosquito vectors are thought to be unprecedented urbanizations combined with inadequate solid waste management, the global movement of people and goods, and most importantly the continuing global climate change [3, 9]. Furthermore, it is also suggested that the global temperature increase may increase the environmental suitability for dengue and other vector-borne arboviral diseases [10].

*Aedes aegypti* is an efficient vector for a number of arboviral diseases [11]. In Ethiopia, *Ae. aegypti* is considered to be a major vector of viral diseases in humans [7, 8]. The species breeds in a variety of man-made water-holding containers such as discarded tyres, mud pots, discarded sinks, polythene sheet, plastic bowl, and buckets [12], sometimes in indoor water storage containers [13, 14] but mainly outdoors [15]. It breeds abundantly during wet seasons and is strongly associated with climatic factors such as rainfall, humidity, and temperature [16]. Abundance of *Aedes* larvae and associated viral diseases transmission risk can be described as house index (HI), breteau index (BI) and container index (CI) [17, 18].

In the absence of effective therapeutic drugs and vaccines against many of the *Aedes*-borne viral diseases, surveillance and control of *Aedes* larvae remains a top priority [19]. Larval control of mosquitoes  is advantageous as adult mosquitoes can fly relatively long distances and survive in a wide range of microhabitats [20]. To implement an effective *Aedes* larvae control strategy, there should be adequate knowledge of its spatio-temporal distribution including habitat location and productivity, seasonal occurrence, habitat type, exposure to sun light, habitat cover and shade, distance from the nearest house, temperature and rainfall [21–24]. In addition, knowledge of chemical characteristics of larval habitats such as pH, conductivity, total alkalinity, hardness, total dissolved solids, dissolved oxygen, and ammonia are also central in prioritizing the larval control and prevention strategies. Although, there have been repeated reports of mosquito-borne viral disease outbreaks in the southern part of the Afar Regional State of Ethiopia, spatio-temporal distribution, habitat characteristics and species composition of *Aedes* larvae remain poorly understood. Thus, this study aimed to assess the spatio-temporal occurrence, habitat characteristics and the species composition of *Aedes* larvae in selected towns of the Afar Regional State, Ethiopia.

## Materials and methods

### Study areas

**Fig. 1:**
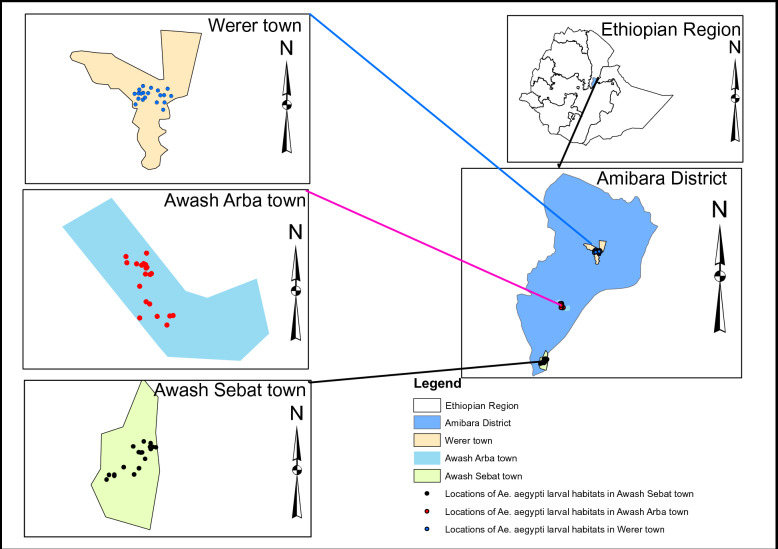
Map of Study areas: Awash Arba, Awash Sebat and Werer Towns, Southern Afar Region, Ethiopia (Source: Ethio_GIS, 2023)

Larvae and pupae of *Aedes* species were surveyed in Awash Arba, Werer, and Awash Sebat towns, Gabi-Rasu Zone, Afar Regional State, Ethiopia from May 2022-April 2023. Awash Arba town is located at about 226 kilo metres (k.m.s) at 9.141111^o^N, 40.15889^o^E and 720–1100 above sea level (m.a.s.l), Werer at 256 kms at 9.33453^o^N, 40.181385^o^E and 720–1100 m.a.s.l and Awash Sebat at 214 km at 8.98810^o^N, 40.163936^o^E and 820–1120 m.a.s.l all to the Northeast of Addis Ababa (Fig. 1). Awash Arba and Werer towns are found in the Amibara district while Awash Sebat town is found in the Awash Sebat administration. Awash Arba and Werer are semi-arid with temperature of 25–35 °C and an average annual rainfall of 530 mm. Awash Sebat experiences 22.6 to 30.6 °C of annual temperature and 606.6 mm of mean annual rainfall [25]. More than 75% of the inhabitants in the towns are pastoralists and agro-pastoralists.

The climate in Afar Regional State is broadly divided into wet and dry seasons. The wet season (locally referred to as Hagaya) the main rainy season of the region spans from June to September while the long dry season (locally known as Gilal) covers the months from October to May [26]. The residents of the three towns store water using man-made containers such as drums, jerrycans and cement tanks at their domestic and peri-domestic areas due to the scarcity of water. Water in the containers was mainly harvested from rainwater and piped water.

### Study design

The three towns were selected purposively in consultation with the local Health Bureaus and on the basis of the recent repeated dengue fever and chikungunya reports. There were 2238 houses in Awash Arba, 3033 in Werer and 3149 in Awash Sebat (Amibara  District and Awash Sebat  Town Health Bureau, 2022, unpublished). Longitudinal prospective study design was used to collect *Aedes* larvae/pupae. A total of 240 surveys were undertaken in each town (20 houses and their environs per month) over a year. To determine the house interval, the total residential houses of each town were divided  by 240 total houses to be surveyed throughout the sampling period. For instance, 2238/240 for Awash Arba, 3033/240 for Werer and 3149/240 for Awash Sebat and gave 9, 12 and 13 intervals for each town respectively. The residential houses were categorized into blocks with the minimum distance between blocks being about 300 meter (m). The blocking of the residential houses was started from the downtown to the peripheral areas to make larval/pupal collection suitable. Thus, the first house was selected randomly from the total of five randomly selected residential houses. The next house was selected systematically using the calculated house interval for each town. To avoid overlapping of the breeding sites among the selected houses and to increase the chances of getting larvae/pupae of *Aedes* species (considering the flight ranges of *Aedes* species); about 300 m distance was added before counting the house interval. *Aedes* larvae/pupae surveys were carried out in relation to each selected house in the following manner. (1) domestic area (inside the house rooms) and outside the house within a radius of about 50 m; (2) Outside the house compound between 50 and 300 m (peri-domestic area) with modification of Minakawa et al. [27].

### *Aedes* larvae/pupae collection and rearing

**Fig. 2:**
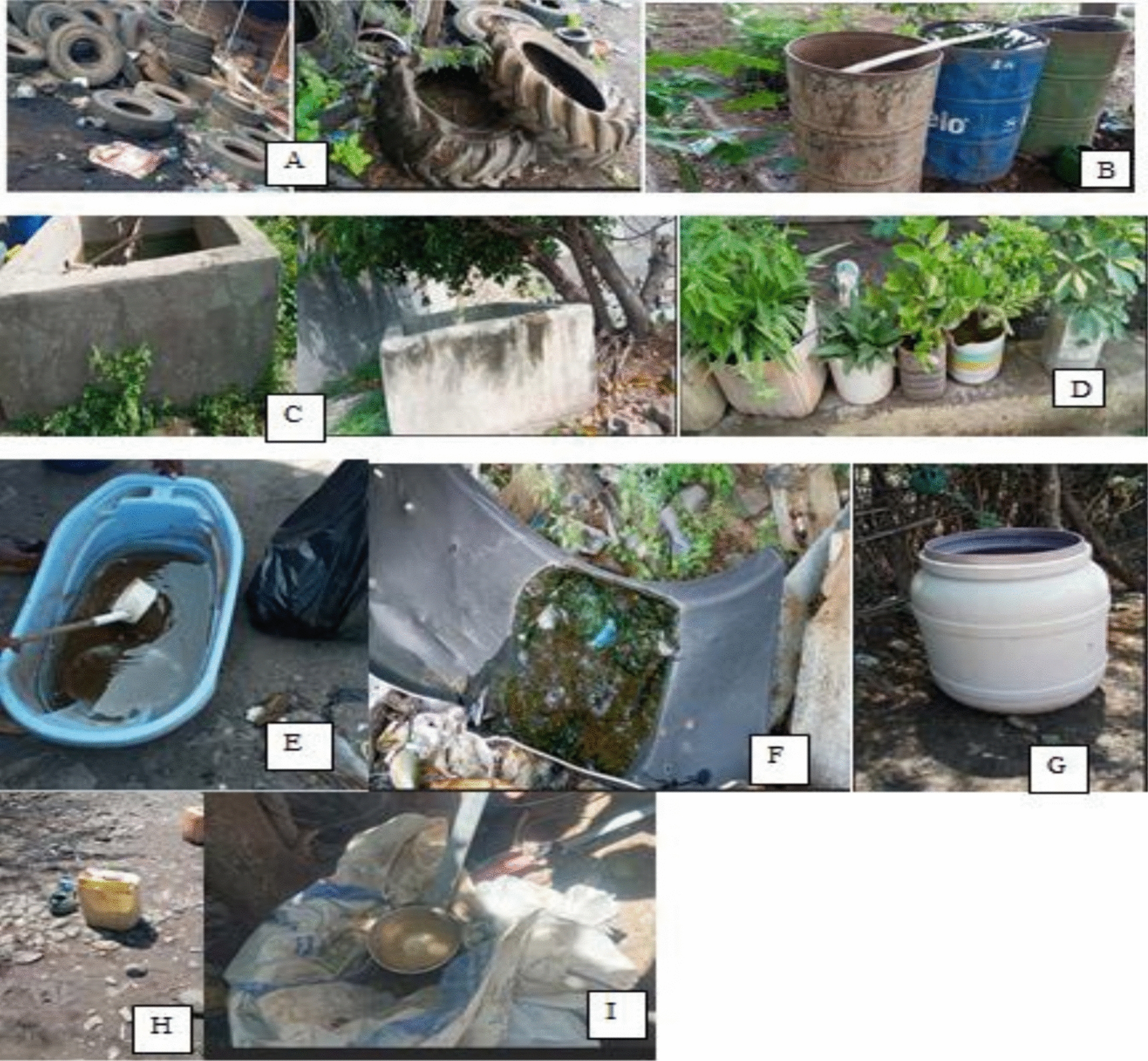
Selected artificial water-holding containers surveyed for *Aedes* larvae/pupae in Awash Arba, Awash Sebat and Werer towns, Ethiopia from May 2022 to April 2023: (**A**) Tyres, (**B**) Water storage drum, (**C**) Water tank made of cement (**D**) Flower pots, (**E**) Plastic bowl (**F**) Discarded plastic, (**G**) Plastic drum, (**H**) Jerrycan, (**I**) Polythene sheet

All available water-holding containers such as used tyres, water storage drums, cement tanks, and flower pots were inspected [28] for the presence of mosquito larvae/pupae and their coordinate readings were recorded using a hand-held global positioning system unit (GPS) (Garmin GPS 60, Garmin international) (Fig. 2). Physical and chemical characteristics of the habitats were recorded during larval collections using a form prepared for the purpose.

A minimum of 10 dips of water samples were collected for larvae/pupae from relatively large water-holding containers using standard dipper (350 ml). Ladles and pipets were used to collect mosquito larvae/pupae from relatively smaller habitats. During the surveys, each water-holding container was classified as positive (if containers harboured at least one mosquito larvae/pupae) or negative (if it did not harbour any mosquito larvae/pupae). Larvae and pupae collected from each positive container were categorized to their respective genus, counted, transferred to a plastic-jar labelled with the date of collection and town, transported to the field insectary in Awash Sebat town where larvae and pupae were transferred to white enamel trays and reared to adults. In the field insectary, larvae in the trays were given powdered fish food (®/TM/©2019 Germany) and covered with netting until they changed to pupae. Then, pupae were transferred to beakers and placed in netting cages (30 × 30 × 30 cm3) with a 10% sugar solution, reared to adults and identified to species morphologically under a standard dissecting microscope following taxonomic keys [29–31].

### Larval and pupal habitat characterisation

Physical habitat characteristics measured larval/pupal habitat location (domestic and peri-domestic), water volume, presence/absence of emerging vegetation, source of water, usage of water, turbidity of water, substrate type, and habitat permanence were recorded. Substrate was categorized as mud, sand, gravel with soil and cement. A larval habitat was considered permanent if it harboured water throughout the year and semi-permanent contained water for approximately 2 to 3 months. Similarly, temporary habitats those which stored water for a short period of time up to 2 to 3 weeks [32]. Distance between water-holding container (habitat) and the nearest house, and between habitat and nearest plant (tree or shrub) were recorded [33]. Habitat temperature was recorded at the time of collection using ordinary thermometer. The turbidity of water was determined as clean or  turbid after taking water samples in glass test tubes and holding them against a white background [27].

Habitat exposure to sunlight was observed visually and recorded as shaded, partially shaded or  exposed fully. The presence of aquatic vegetation was observed and recorded as present or absent. Distance of larval/pupal habitat to the nearest house was measured using a tape (meter). About 250 ml water was collected from larvae/pupae positive habitats using polyethylene bottles, transported with cold boxes and analysed for habitat chemical characteristics such as alkalinity, salinity, conductivity, total dissolved solids, dissolved oxygen and total hardness at the Chemistry Department, Addis Ababa University with the permission of the Africa Center of Excellence for Water Management, using a standard method of water examination [34].

### Statistical analysis

Data were analysed using SPSS version 20. Mean larval/pupal density of *Ae. aegypti* for each container was calculated by dividing the total number of larvae or pupae to the number of dips. Prior to data analysis, data were log-transformed [log (n + 1)] to fit the normal distribution curve and checked for normality by the Shapiro–Wilk test. Independent T test was used to determine the mean density of *Ae. aegypti* between two groups of physical characteristics of larval habitats and One-way analysis of variance (ANOVA) was used to compare mean larvae/pupae densities of *Ae. aegypti* of more than two groups of physical characteristics among the habitats. When significant differences were observed in using ANOVA, Tukeyʼs post-hoc test was used for pairwise comparisons of the means [35]. Pearson correlation was used to assess the correlations of *Ae. aegypti* larvae/pupae and habitat chemical characteristics. Bivariate analysis was performed to assess associations between habitat positivity for *Ae. aegypti* larvae/pupae and physical characteristics of larval habitats. Then, multiple logistic regression was carried out to determine key predictors. The odds ratio (OR), 95% confidence intervals (95% CI) and p value were determined. In addition, multiple linear regression was used to assess the relations between *Ae. aegypti* larvae/pupae density and the habitat chemistry of water-holding containers. All *p* values < 0.05 were considered statistically significant. The larval/pupal infestation level was computed using indices namely, House index (HI), Container index (CI), Breteau index (BI) and Pupal index (PI) [36].

$${\text{House index}}\, = \,\frac{{{\text{Number}}\,{\text{of}}\,{\text{positive}}\,{\text{house}}}}{{{\text{Number}}\,{\text{of}}\,{\text{house}}\,{\text{inspected}}}}\, \times \,100$$

$${\text{Container index}}\, = \,\frac{{{\text{Number}}\,{\text{of}}\,{\text{positive}}\,{\text{containers}}}}{{{\text{Number}}\,{\text{of}}\,{\text{containers}}\,{\text{inspected}}}}\, \times \,100$$

$${\text{Breteau index}}\, = \,\frac{{{\text{Number}}\,{\text{of}}\,{\text{positive}}\,{\text{containers}}}}{{{\text{Number}}\,{\text{of}}\,{\text{house}}\,{\text{inspected}}}}\, \times \,100$$

$${\text{Pupal index}} \, = \,\frac{{{\text{Number}}\,{\text{of}}\,{\text{pupae}}}}{{{\text{Number}}\,{\text{of}}\,{\text{house}}\,{\text{inspected}}}}\, \times \,100$$

## Results

### Occurrence of *Aedes aeygpti* larvae/pupae

All of the *Aedes* larvae/pupae that emerged to adults were morphologically identified as *Aedes aegypti* and hence all *Aedes* larvae and pupae collections are hereafter considered to be *Ae. aegypti.* A total of 11,440 larvae/pupae were collected, of which 79.5% (*n* = 9099) were *Ae. aegypti*. Among the 9099 larvae/pupae, 53.6% (*n* = 4875) were collected from Awash Sebat, 29.5% (*n* = 2687) from Awash Arba, and 16.9% (*n* = 1537) from Werer (Table 1). A total of 1544 *Culex* larvae/pupae were collected, among which 47.99% (*n* = 741) were from Werer, 41.6% (*n* = 643) from Awash Sebat, and 10.36% (*n* = 160) from Awash Arba. Moreover, 797 *Anopheles* larvae/pupae were collected, of which 76% (*n* = 606) were from Awash Sebat, 20.3% (*n* = 162) from Awash Arba, and 3.6% (n = 29) from Werer towns (Table 1).

**Table 1:** Mosquito larvae/pupae collected from water-holding containers from Awash Sebat, Awash Arba and Werer towns of Afar Regional States, Ethiopia, May 2022- April 2023

			Mosquito genera/species			
Study sites	THS (+ ve)	TCI(+ ve)	*Ae. aegypti *n(%)	*Anopheles n*(%)	*Culex* *n* (%)	Total *n* (%)
Awash Sebat	240 (93)	375 (275)	4875 (53.56)	606 (76.04)	643 (41.65)	6124 (53.53)
Awash Arba	240 (45)	142 (121)	2687 (29.54)	162 (20.32)	160 (10.36)	3009 (26.30)
Werer	240 (21)	82 (62)	1537 (16.9)	29 (3.64)	741 (47.99)	2307 (20.17)
Total	720 (159)	599 (459)	9099 (100)	797 (100)	1544 (100)	11,440 (100)

TCI, Total containers Inspected THS, Total Houses Surveyed; + ve, Positive houses and containers

### Productivity of *Ae. aegypti* larvae/pupae with container types and location

In Awash Arba town, 97.1% (2610/2687) of the *Ae. aegypti* larvae/pupae were collected from domestic sites and 2.9% (77/2687) from the peri-domestic sites. Similarly, in Werer, 91.8% (1411/1537) of the total *Ae. aegypti* larvae/pupae collections were from domestic sites and 8.2%(126/1537) from the peri-domestic sites. Likewise, in Awash Sebat town, 72.5% (3533/4875) of the total *Ae. aegypti* catches were made from domestic and 27.5% (1342/4875) from peri-domestic sites (Table 2).

**Table 2:** Container types, location and level of *Ae. aegypti* infestation in Awash Sebat, Awash Arba and Werer towns of Afar regional states of Ethiopia, May 2022 to April 2023

Study sites	Container location	Container types	Containers inspected (positive)	*Ae. aegypti n* (%)
Awash Sebat	Domestic	Tyres	98 (83)	1488 (30.5)
		Drums	31 (22)	1011 (20.7)
		Flower pots	14 (10)	164 (3.4)
		Discarded plastics	9 (6)	106 (2.2)
		Others(cement–water tank, bowl, jerrycan)	46 (36)	764 (15.7)
	Peri-domestic	Tyres	127 (79)	992 (20.3)
		Drums	8 (4)	83 (1.7)
		Flower pots	0 (0)	0 (0)
		Discarded plastics	0 (0)	0 (0)
		Others	42 (20)	267 (5.5)
		Sub-total	375 (260)	4875 (100)
Awash Arba	Domestic	Tyres	95 (69)	1974 (73.5)
		Drums	33 (18)	572 (21.3)
		Flower pots	0 (0)	0 (0)
		Discarded plastics	2 (1)	44 (1.6)
		Others	3 (1)	20 (0.7)
	Peri-domestic	Tyres	9 (8)	77 (0.8)
		Drums	0 (0)	0 (0)
		Flower pots	0 (0)	0 (0)
		Discarded plastics	0 (0)	0 (0)
		Others	0 (0)	0 (0)
		Sub-total	142 (97)	2687 (100)
Werer	Domestic	Tyres	53 (37)	1351 (87.9)
		Drums	3 (2)	0 (0)
		Flower pots	0 (0)	0 (0)
		Discarded plastics	0 (0)	0 (0)
		Others	18 (8)	60 (3.9)
	Peri-domestic	Tyres	8 (6)	126 (8.2)
		Drums	0 (0)	0 (0)
		Flower pots	0 (0)	0 (0)
		Discarded plastics	0 (0)	0 (0)
		Others	0 (0)	0 (0)
		Sub-total	82 (5)	1537 (100)
	Overall total	Domestic	405 (294)	7554/9099
		Peri-domestic	194 (116)	1545/9099

The highest *Ae. aegypti* larvae and pupae collections in all the towns was made from water-holding tyres. In Werer, domestic water-holding tyres harboured 87.9% (1351/1537) of *Ae. aegypti* larvae/pupae collections and 73.5% (1974/2687) in Awash Arba. In the domestic sites of Awash Sebat town, 30.5% (1488/4875) of the *Ae. aegypti* were collected from water-holding tyres, 20.7% (1011/4875) from water-storage drums, and 15.8% (764/4875) from water tanks made of cement. Discarded plastic contributed 2.2% (106/4875) of the *Ae. aegypti* larvae/pupae in Awash Sebat and 1.6% (44/4875) in Awash Arba whereas flowerpots had 3.3% (164/4875) in the domestic areas of Awash Sebat (Table 2).

### Larval/pupal indices

Overall, 720 surveys were made during the 12 months, of which 460 resulted in at least one positive water-holding container for *Ae. aegypt, Anopheles* or *Culex* larvae/pupae (Table 3)*.* 15.8% (*n* = 114) of the surveys were associated with *Ae. aegypti* larvae/pupae in domestic and peri-domestic sites. The container indices (CIs) ranged from 8.3 to 85.3% in Awash Sebat, 5 to 91.8% in Awash Arba and 33.3 to 88.4% in Werer. The House Indices (HIs) were also from 5–80% in Awash Sebat, 5–75% in Awash Arba and 5–35 in Werer. Similarly, Breteau Indices (BIs) were 5–405 in Awash Sebat, 5–225 in Awash Arba and 10–190 in Werer. The highest CIs of Awash Sebat (85.3%) and Awash Arba (75%) were observed in September 2022 and that of Werer (35%) in August 2022. In addition, relatively higher House Index (80%) was observed in Awash Sebat during August, as compared to Awash Arba (55%) and Werer (35%) sites. Moreover, higher Breteau Index (405) and Pupal Index (1320) were recorded in Awash Sebat in September than Werer and Awash Arba areas.

**Table 3:** *Aedes aegypti* larval/pupal indices in Awash Sebat, Awash Arba and Werer towns of Afar regional state of Ethiopia May 2022 to April 2023

Study sites	Study period	Positive houses	Total houses	Total containers	Positive containers	No. pupae	Indexes			
							Cl (%)	HI (%)	BI	PI
Awash Sebat	Jun2022	2	20	18	4	33	22.22	10	20	165
	Jul 2022	6	20	32	9	71	28.1	30	45	355
	Aug2022	16	20	116	69	170	59.5	80	345	850
	Sept2022	14	20	95	81	264	85.3	70	405	1320
	Oct 2022	14	20	86	64	131	74.4	70	320	655
	Nov2022	3	20	8	4	9	50	15	20	45
	Dec2022	2	20	12	3	6	25	10	15	30
	Jan 2023	2	20	3	2	1	66.6	10	10	5
	Feb 2023	1	20	2	1	2	50	5	5	10
	Mar2023	1	20	2	1	3	50	5	5	15
	Apr 2023	1	20	2	1	7	50	5	5	35
	Total	63	240	375	240	699	87.6	26.25	100	291.25
Awash Arba	May2022	0	20	0	0	0	0	0	0	0
	June2022	0	20	0	0	0	0	0	0	0
	Jul 2022	6	20	15	10	0	66.6	30	50	0
	Aug2022	11	20	39	30	89	76.9	55	150	445
	Sept2022	15	20	49	45	89	91.8	75	225	445
	Oct2022	8	20	30	24	83	80	40	120	415
	Nov2022	1	20	2	1	4	50	5	5	20
	Dec2022	2	20	6	3	0	50	10	15	0
	Jan 2023	1	20	2	1	0	5	50	5	25
	Feb 2023	0	20	0	0	0	0	0	0	0
	Mar2023	0	20	0	0	0	0	0	0	0
	Apr 2023	0	20	0	0	0	0	0	0	0
	Total	44	140	143	114	265	35.03	22.08	47.5	112.5
Werer	May2022	0	20	0	0	0	0	0	0	0
	Jun2 022	0	20	0	0	0	0	0	0	0
	Jul 2022	0	20	0	0	0	0	0	0	0
	Aug2022	7	20	43	38	123	88.4	35	190	615
	Sept2022	6	20	24	14	136	58.3	30	70	680
	Oct 2022	1	20	6	2	5	33.3	5	10	25
	Nov2022	0	20	0	0	0	0	0	0	0
	Dec2022	1	20	4	2	5	50	5	10	25
	Jan 2023	0	20	0	0	0	0	0	0	0
	Feb 2023	0	20	0	0	0	0	0	0	0
	Mar2023	0	20	0	0	0	0	0	0	0
	Apr 2023	0	20	0	0	0	0	0	0	0
	Total	15	80	77	56	269	19.2	6.25	23.33	112.08
Overall total		114	460	599	410	1233	47.28	18.19	59.94	171.94

HI, House Index; Cl, Container index; BI, Breteau Index, PI, Pupal Index

### Spatial and temporal distribution of *Ae. aegypti* larvae/pupae

**Fig. 3:**
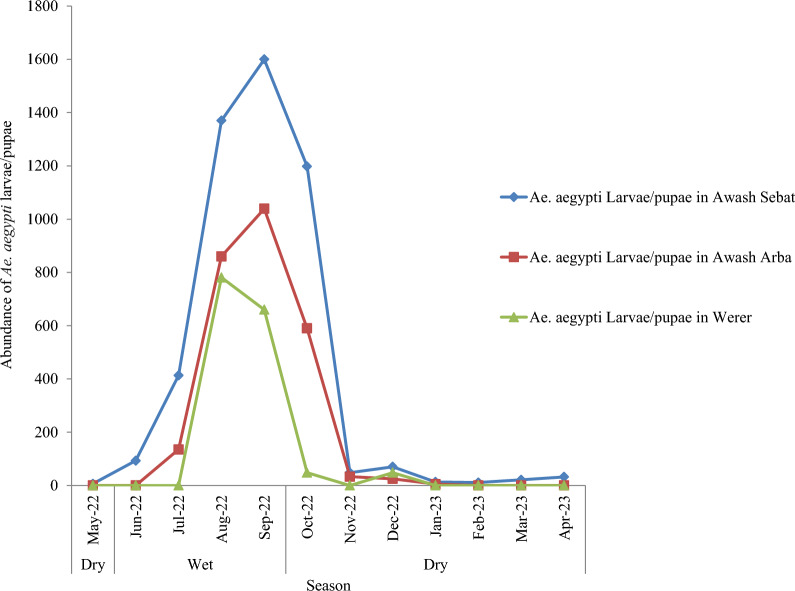
Spatial and temporal distribution of *Ae. aegypti* larvae/pupae in Awash Sebat, Awash Arba and Werer towns of Afar regional state of Ethiopia from May 2022 to April 2023

The spatio-temporal distribution of *Ae. aegypti* larvae/pupae varied between the dry and wet season in the study towns. The peak *Ae.aegypti* larvae/pupae collections in Awash Sebat and in Awash Arba towns were made during September 2022 and in Werer town during August 2022 (Fig. 3).

### Association of *Ae. aegypti* larval/pupal density and occurrence with physical characteristics

The results of mean comparisons of the physical characteristics and densities of the *Ae. aegypti* larvae/pupae were depicted in Table 4. Significantly higher mean densities of *Ae. aegypti* larvae/pupae were collected during the wet season (*F* = 15.075, *p* < 0.001), from tyre habitat types (*F* = 4.775, *p* = 0.001), habitats with gravel with soil substrate (*F* = 7.085, *p* < 0.001) and rain water sources (*F* = 6.020, *p* = 0.003). Significant differences were observed in *Ae. aegypti* mean larval density with substrate types. Further, Tukeyʼs post-hoc test indicated that water-containers with mud and gravel with soil substrates had  significantly higher *Ae. aegypti* larval/pupal densities compared to cement substrates (*p* < 0.001). In addition, significant mean differences were observed between tap and rain water sources (*p* = 0.002).

**Table 4:** Physical characteristics of water-holding containers and mean density of *Ae. aegypti* larvae/pupae in Awash Sebat, Awash Arba and Werer towns, Afar regional state, Ethiopia, May 2022 to April 2023

Characteristics	Variables	Mean ± SE F *P*-value		
Season	Dry	1.47 ± 0.07	15.075	< 0.001
	Wet	1.84 ± 0.03		
Substrate types	Mud	1.75 ± 0.05	7.085	< 0.001
	Sand	1.59 ± 0.13		
	Gravel with soil	1.76 ± 0.05		
	Cements	1.09 ± 0.12		
Sun light Exposure	Shaded	1.75 ± 0.11	0.125	0.883
	Partially shaded	1.71 ± 0.04		
	Open	1.67 ± 0.07		
Habitat permanency	Semi-permanent	1.67 ± 0.09	2.493	0.462
	Temporary	1.74 ± 0.04		
Habitat type	Flower pots	1.42 ± 0.25	4.775	0.001
	Drums	1.56 ± 0.08		
	Discarded plastics	1.43 ± 0.11		
	Tyres	1.84 ± 0.03		
	Others(Cement tank)	1.56 ± 0.14		
Turbidity	Clean	1.60 ± 0.07	8.299	0.043
	Turbid	1.77 ± 0.03		
Water usage	Sometimes	1.65 ± 0.05	5.311	0.136
	Not used	1.77 ± 0.04		
Water source	Tap water	1.52 ± 0.08	6.020	0.003
	Rain water	1.82 ± 0.03		
	Mixed water	1.75 ± 0.09		
Habitat location	Domestic	1.86 ± 0.08	0.338	0.126
	Peri-domestic	1.70 ± 0.03		
Presence of vegetation	Present	1.72 ± 0.06	0.006	0.823
	Absent	1.70 ± 0.04		

SE, Standard error

The bivariate analysis revealed that containers located at domestic sites, rain water source, shaded and partially shaded habitats, water-holding tyres, water storage drums, mud and gravel with soil substrates were significantly associated with the occurrences of *Ae. aegypti* larvae/pupae. On the other hand, dry season was less likely to harbour *Ae. aegypti* (Additional file 1). Moreover, the multiple logistic regression analysis showed that occurrences of *Ae. aegypti* larvae/pupae were more likely found in tyres (AOR = 15.89, CI = 3.55–71.09, *p* < 0.001) and water storage drums (AOR = 19.84, CI = 4.64–84.89, *p* < 0.001) as compared to other habitats. In addition, containers located in domestic sites were 3.76 times more likely to harbour *Ae. aegypti* larvae/pupae as compared to peri-domestic sites (AOR = 3.76, CI = 1.27–11.12, *p* = 0.017) and habitats with tap water source were 0.08 times less likely harboured *Ae. aegypti* larvae/pupae as compared to mixed water source (AOR = 0.08, CI = 0.02–0.31, *p* = 0.001) (Table 5).

**Table 5:** Relationship between *Ae. aegypti* larvae/pupae occurrences and larval habitat physical characteristics in Awash Sebat, Awash Arba and Werer towns of Afar regional states of Ethiopia, May 2022 to April 2023

Habitat characteristics	Variables	AOR (95% CI)	*p*-value
Season	Wet	1	
	Dry	0.60 (0.24–1.46)	0.260
Water source	Mixed	1	
	Tap	0.08 (0.02–0.31)	< 0.001
	Rain	1.48 (0.10–2.27)	0.353
Substrate types	Cement	1	
	Mud	0.86 (0.19–3.72)	0.837
	Sand	0.70 (0.08–6.29)	0.752
	Gravel with soil	1.03 (0.32–3.34)	0.961
Sun light exposure	Exposed fully	1	
	Shaded	5.33 (0.57–49.47)	0.141
	Partially shaded	0.47 (0.13–1.61)	0.227
Habitat types	Other (water tank made of cement)	1	
	Drum	19.84 (4.64–84.89)	< 0.001
	Discarded plastics	2.65 (0.18–38.23)	0.474
	Tyres	15.89 (3.55–71.09)	< 0.001
Habitat location	Peri-domestic	1	
	Domestic	3.76 (1.27–11.12)	0.017

AOR, Adjusted odds ratio; CI, Confidence interval

### Relation of *Aedes* aegypti larval/pupae density with larval habitat chemistry

Positive correlations were observed between *Ae. aegypti* larvae/pupae densities and habitat chemistries including water temperature, total alkalinity, total hardness, electrical conductivity, total dissolved solids, dissolved oxygen and salinity (Additional file 2). Moreover, further multiple linear regression revealed that *Ae. aegypti* larvae/pupae densities showed positive relations with dissolved oxygen (β= 0.523, *p* < 0.001) and total hardness (β = 0.475, *p* = 0.034) of the water (Table 6). On the other hand, water temperature seemed to positively influence density of *Ae. aegypti* larvae/pupae but the relation was not statistically significant. Similarly, salinity of water-holding containers was negatively related even if its effect was not statistically significant.

**Table 6:** Relationship between habitat chemistry and *Ae. aegypti* larval/puae density in Awash Sebat, Awash Arba and Werer towns of Afar regional states of Ethiopia, May 2022 to April 2023

Variables	Standard error	Beta	t	*p*-value
Water temperature(^o^C)	0.017	0.130	1.506	0.135
Total hardness(mg/l)	0.002	0.475	1.890	0.034
Dissolved oxygen(mg/l)	0.028	0.523	4.077	< 0.001
Salinity (%)	0.675	− 0.494	− 1.714	0.089

R2 = 0.289; Adjusted R2 = 0.262; F (10.583)

## Discussion

The recent outbreaks of dengue fever and chikungunya virus in the Eastern and North-eastern parts of Ethiopia including the Afar Region became a public health concern [5, 7, 8]. As the majority of such arboviral diseases lack effective therapeutic treatment and vaccines, managing *Aedes* species that transmit the diseases is the preferred controlling strategy. In Ethiopia, limited studies were conducted on larval habitats of *Aedes* mosquitoes with a few months of collections which could overlook the most prolific breeding time [12, 37]. Thus, longitudinal studies to understand the distribution and habitat characterization of the *Aedes* mosquito remain poorly investigated in the country. This necessitated a longitudinal study of *Aedes* species larval habitats in time and space in the Southern part of Afar region. The study explored diverse *Ae. aegypti* larvae/pupae habitats and characterized them. Thus, the findings serve as a baseline data to the targeted *Aedes* mosquito control interventions and to minimize the risks of *Aedes*-transmitted viral diseases.

*Aedes aegypti* larvae/pupae were found most abundantly in water-holding tyres in Awash Sebat, Awash Arba and Werer towns of Southern Afar. This was in agreement with previous studies in Dire Dawa City administration [12, 38], in Metema and Humera areas of Northwest Ethiopia [37], and in Kebridehar town of Somalia Regional State [39]. Similar results were also reported from Malaysia [40]. However, the study conducted in Zanzibar city of Tanzania, showed discarded plastics and metals as preferred habitats  for *Ae. aegypti* larvae [41]. Tyres as a major *Ae. aegypti* larval habitat in the present study towns could be due to their low level of disturbance, they provide shade and protection for both larvae and adults [42]. For instance, in the current study, *Ae. aegypti* larvae/pupae densities were highest in shaded larval habitats, and shade of breeding habitats was positively correlated to mosquito larvae/pupae as previously indicated [43]. Thus, proper management of water-holding tyres including other potential habitats should be implemented to reduce the breeding of *Ae. aegypti*.

Besides to tyres, water storage drums were also observed to be the second *Ae. aegypti* larval/pupal habitats. Our finding of water storage drums as a breeding habitat of *Ae. aegypti* was comparable with the reports from different parts of the world including Mozambique and Nicaragua of America [44, 45]. In the study towns, the households store water with drums for drinking and domestic use or for house construction purposes. Improper storages of water with domestic containers like drums may serve as the breeding ground for a  possible infestation with *Ae. aegypti* larvae/pupae as previously reported [46]. Thus, adequate use of water storage drums could be a strategy to control *Ae. agypti* larvae/pupae.

About 83% (7554/9099) of *Ae. aegypti* larvae/pupae collections were made from the domestic sites of residential houses while the 17% (1545/9099) were from the peri-domestic sites. The abundance of *Ae. aegypti* larvae/pupae mostly close to human habitations in domestic and peri-domestic breeding habitats were also reported previously [47]. However, no *Ae. aegypti* larvae/pupae were collected from inside the house rooms. This result was contrary to previous research findings from Sudan and in Western and coastal Kenya in that they reported *Ae. aegypti* larvae and pupae from inside residential rooms [19, 48]. The absence of *Ae. aegypti* larvae/pupae inside the house rooms in the present survey could be due to the fact that the hygienic condition of water-holding containers found inside the house rooms which were cleaned by the households frequently. They were also covered which make the containers being unproductive to *Aedes* mosquito immature as previously reported [49]. Overall, the collection of *Ae. aegypti* larvae/pupae in higher number and proportions were from the domestic sites followed by peri-domestic sites. Thus, emphasis should be given to domestic sites followed by the peri-domestic sites in surveying and managing *Ae. aegypti* larvae/pupae in the study areas.

Estimating *Aedes* species infestation status using indices like CI, HI, and BI is important to measure the success of vector control strategies [50]. The CI, HI, and BI values observed during the wet season, especially in the months of July 2022 to October 2022 were exceeded 5% in the study towns. The BI and HI values observed were also higher than the findings from the previous studies from Tanzania [51] and Cameroon [52]. However, the average CI, HI, and BI reported in the present study were relatively lower than indices previously reported from Dire Dawa City administration [12]. Thus, index values of *Ae. aegypti* species estimated, were high compared to the World Health Organization epidemic thresholds of transmission risk established for yellow fever [53], for dengue and other arboviruses [54]. The high infestation indices of *Ae. aegypti* suggest a risk for large outbreaks of arbovirus infections such as dengue, yellow fever and chikungunya in the Southern Afar Region of Ethiopia. Thus, *Aedes* species habitat management practices should be implemented in the study area to reduce the *Aedes* mosquito density and prevention of arboviral diseases outbreaks.

The average pupae indices (PIs) observed in the present study were higher compared to the Bacongo and the M’flou areas of Republic of Congo [50]. However, lower than from previous study in Dire Dawa City administration, Ethiopia [38]. The pupal index was included in the study as it has more epidemiological significance [28]. Including pupal indices in  the *Aedes* species breeding sites preference is important to quantify *Aedes* species infestation and predicts epidemiological risks as it gives numeric figures by dividing number of pupae in each site per hectar, per houses and per person [55]. In addition, pupal indices are also vital since the relationship  between pupal densities and adult densities are usually directly proportional [56].

The results from the analysis of variance (ANOVA) indicated that the mean density of *Ae. aegypti* larvae/pupae were influenced by the physical characteristics such as wet season, turbid water, gravel with soil substrate type, rain water source, and tyre habitat type. Similarly, the abundances of *Ae. aegypti* larvae/pupae were increased from August 2022 to October 2022 then declined towards April 2023 in all the study towns. The results suggest that the wet season contributed to the increased in the larvae/pupae density and abundance. Similar findings were observed in Tanzania, where more *Ae. aegypti* larvae collections were undertaken during the wet season [51]. Wet season as major *Ae. aegypti* larvae collection in the present study areas could be due to the fact that the increase in the number of water-holding containers as a result of the availability of local rainfall, temperature, and humidity of the study sites. For instance, the Afar Regional State received a large amount of rainfall during the months of June to early October and the rest of the months are dry [26]. Thus, particular emphasis should be given to apply the larval source management especially during the wet season in the study areas [57]. However, the *Ae. aegypti* larvae/pupae collected during the dry season were  not undermine since breeding sites present during dry season serve as a reservoir which may have an impact on the spread of mosquito-borne diseases during the wet season [58].

The types of substrate present in the water-holding containers was also the determinant factor to the density of *Ae. aegypti* larvae/pupae. The highest *Ae. aegypti* larvae/pupae density was observed in gravel with soil substrate than other water-holding containers. This may be due to differences in the organic content of the substrates of breeding containers. In the current study, higher density of *Ae. aegypti* larvae/pupae were also associated with turbid water than the clean water. In-line with this, the study conducted in the Central African Republic observed that *Ae. aegypti* larvae/pupae were observed in turbid water [33]. However, contrary to the present finding, the study conducted in Tanzania reported that higher *Ae. aegypti* immatures were collected from containers with clean water than turbid water[51]. The existence of high density of *Ae. aegypti* larvae in turbid water in the present study may be due to turbid water may contained detritus which serve as food to the larvae or it also aid to prevent the larvae from aquatic predators by hiding them [33].

Further, the multiple logistic regression analysis identified key predictors including tyres and water storage drums, domestic sites and tap water source for the occurrences of *Ae. aegypti* larvae/pupae. For instance, water-holding tyres and water storage drums were significantly associated with *Ae. aegypti* larvae/pupae occurrence. Similar findings were reported in Zanzibar, tyre as a major risk factor for the occurrence of *Ae. aegypti* larvae/pupae habitat [59]. Tap water source of the habitats was negatively associated to the occurrences of *Ae. aegypti* larvae/pupae. This was in agreement with the study conducted in Brazzaville Congo [50]. In addition, the occurrence of *Ae. aegypti* larvae/pupae was more significantly associated with containers located in domestic sites than in the peri-domestic sites. This could be due to the existence of domestic containers for longer period in the study areas.

The multiple linear regression result revealed that significantly positive relations were observed between *Ae. aegypti* larvae/pupae density and dissolved oxygen in  water-holding containers. Similar previous findings were observed in the Kinshasa area of Democratic Republic Congo [60]. Moreover, *Ae. aegypti* larvae/pupae densities were also positively related with total hardness of water-holding containers. This result was in agreement with  the study conducted in Northern Iran, where total hardness of the breeding containers had a positive relations  on increase larval density [61]. On the other hand, water temperature seemes to positively influence density of *Ae. aegypti* larvae/pupae but the relation was not statistically significant. Similarly, salinity of water-holding containers was negatively related even if its effect was not statistically significant. Thus, understanding the correlations among larval habitat characteristics and *Ae. aegypti* larval/pupal densities suggest the effect of these factors on the productivity of *Ae. aegypti* and  which in turn can be used to proposed future control and management strategies for  *Aedes*-borne diseases.

### Limitations of the study

The study mainly considered characterizing the abiotic factors (physicochemical characteristics) of *Ae. aegypti* larvae. Thus, further research should be inspired to include the biological entities influencing the density of *Ae. aegypti* larvae/pupae in their breeding habitats. In addition, research should also extend to investigate the physicochemical parameters since we measured these parameters during the wet season. Molecular identification of *Ae. aegypti* species and sub-species were not performed due to logistics constraints such as the unavailability of primers and reagents.

## Conclusion

*Aedes aegypti* larvae and pupae occurred in a wide range of water-holding containers in domestic and peri-domestic areas. The major *Ae. aegypti* larvae/pupae habitats were water-holding used tyre, water storage drums, water tanks made of cement, flowerpots, and discarded plastics. The productivity of *Ae. aegypti* larval habitats were higher in tyres and drums habitats, which were located at domestic sites. In addition, dissolved oxygen and total hardness of the containers influence *Ae. aegypti* larval/pupal productivity. The values of larval indices (Container index, House index and Breteaux index) observed were highly indicators to undergo vector control campaigns to control *Aedes*-borne diseases and their potential outbreaks in the study areas. Thus, data recorded in the present could serve as baseline to implement vector control programmes, especially in designing of larval sources management in the study areas. Further studies in different zones of the Afar region and Ethiopia should be inspired, where arboviral diseases outbreak experienced with a special focus on the biotic and other abiotic factors in both productive and non-productive larval habitats towards a sound understanding of *Ae. aegypti* larval ecology and application of appropriate larval control measures.

### Supplementary Information

Additional file 1: Table S1. Bivariate analysis of the relationship between *Ae. aegypti* larvae/pupae occurrences and larval habitat physical characteristics in Awash Sebat, Awash Arba and Werer towns of Afar regional states of Ethiopia, May 2022 to April 2023.

Additional file 2: Table S2. Correlation coefficients between habitat chemistry and* Ae. aegypti* larvae/pupae density in Awash Arba, Awash Sebat and Werer towns of Afar Region, Ethiopia, May 2022 to April 2023.

## Data Availability

All data generated or analysed to support the findings in present study are included in the manuscript and its additional files.
