# Supplementary material for: Spatio-temporal occurrence and habitat characteristics of Aedes aegypti (Diptera: Culicidae) larvae in Southern Afar region, Ethiopia
Source: Trop Med Health. 2024 Aug 2;52:51. doi: 10.1186/s41182-024-00612-5 (PMC11295501; doi:10.1186/s41182-024-00612-5)
Supplement: Supplementary file 1 — Additional file 1: Table S1. Bivariate analysis of the relationship between Ae. aegypti larvae/pupae occurrences and larval habitat physical characteristics in Awash Sebat, Awash Arba and Werer towns of Afar regional states of Ethiopia, May 2022 to April 2023. [file 41182_2024_612_MOESM1_ESM.docx]

| Habitat characteristics | Variables | COR (95% CI) | p-value |
| --- | --- | --- | --- |
| Season | Wet | 1 |  |
|  | Dry | 0.41 (0.28-0.58) | <0.001 |
| Water source | Mixed | 1 |  |
|  | Tap | 0.818 (0.53-1.27) | 0.372 |
|  | Rain | 4.87 (2.79-8.48) | <0.001 |
| Substrate types | Cement | 1 |  |
|  | Mud | 1.9(1.14-3.19) | 0.014 |
|  | Sand | 1.4(0.44-4.41) | 0.566 |
|  | Gravel with soil | 1.8(1.21-2.71) | 0.004 |
| Sun light exposure | Exposed fully | 1 |  |
|  | Shaded | 4.00(1.13-14.17) | 0.032 |
|  | Partially shaded | 1.86(1.32-2.62) | <0.001 |
| Habitat types | Other (water tank made of cement) | 1 |  |
|  | Drum | 7.0(2.45-19.95) | <0.001 |
|  | Discarded plastics | 2.50(0.48-12.88) | 0.273 |
|  | Tyres | 7.10(3.66-13.76) | <0.001 |
| Habitat location | Peri-domestic | 1 |  |
|  | Domestic | 2.63(1.84-377) | <0.001 |
| Habitat permanency | Semi-permanent | 1 |  |
|  | Temporary | 2.44(1.13-5.31) | 0.124 |
| Water turbidity | Clear | 1 |  |
|  | Turbid | 1.53(.98-2.39) | 0.061 |
| Water usage | Sometimes | 1 |  |
|  | Not used | 1.14(0.78-163) | 0.469 |

COR: Crude odds ratio; CI: Confidence interval
