# Supplementary material for: Spatio-temporal occurrence and habitat characteristics of Aedes aegypti (Diptera: Culicidae) larvae in Southern Afar region, Ethiopia
Source: Trop Med Health. 2024 Aug 2;52:51. doi: 10.1186/s41182-024-00612-5 (PMC11295501; doi:10.1186/s41182-024-00612-5)
Supplement: Supplementary file 2 — Additional file 2: Table S2. Correlation coefficients between habitat chemistry and Ae. aegypti larvae/pupae density in Awash Arba, Awash Sebat and Werer towns of Afar Region, Ethiopia, May 2022 to April 2023. [file 41182_2024_612_MOESM2_ESM.docx]

| Chemical variables | *Ae. aegypti* |
| --- | --- |
| Water temperature(^o^C) | 0.217* |
| pH | 0.046 |
| Total alkalinity(mg/l) | 0.456** |
| Total hardness(mg/l) | 0.416** |
| Salinity(%) | 0.427** |
| Conductivity((μS/cm) | 0.436** |
| Total dissolved solids(mg/l) | 0.436** |
| Dissolved oxygen(mg/l) | 0.527** |

** The correlation was significant at 0.001 levels (two tailed)

*The correlation was significant at 0.01 levels (two tailed
